# Evaluation of Locally Isolated Entomopathogenic Fungi against Multiple Life Stages of *Bactrocera zonata* and *Bactrocera dorsalis* (Diptera: Tephritidae): Laboratory and Field Study

**DOI:** 10.3390/microorganisms9081791

**Published:** 2021-08-23

**Authors:** Muhammad Usman, Waqas Wakil, Jaime C. Piñero, Shaohui Wu, Michael D. Toews, David Ian Shapiro-Ilan

**Affiliations:** 1 Department of Entomology, University of Agriculture, Faislabad 38040, Pakistan; usmanbhattiuaf@gmail.com; 2 Senckenberg German Entomological Institute, D-15374 Müncheberg, Germany; 3 Stockbridge School of Agriculture, University of Massachusetts, Amherst, MA 01003, USA; jpinero@umass.edu; 4 Department of Entomology, University of Georgia, Tifton, GA 31793, USA; shaohui.wu@uga.edu (S.W.); mtoews@uga.edu (M.D.T.); 5 USDA-ARS, SE Fruit & Tree Nut Research Laboratory, Byron, GA 31008, USA

**Keywords:** entomopathogenic fungi, horizontal transmission, host development, biological control

## Abstract

Fruit flies including *Bactrocera zonata* and *B. dorsalis* (Diptera: Tephritidae) are considered major pests of orchard systems in Pakistan. This study evaluated the laboratory virulence, sub-lethal effects, horizontal transmission, greenhouse, and field-cage efficacy of locally isolated entomopathogenic fungi (EPF) against *B. zonata* and *B. dorsalis*. In virulence assays against third instars and adults, all 21 EPF isolates (*Beauveria bassiana* and *Metarhizium anisopliae*) tested were pathogenic and caused varying levels of mortality to the fruit flies. Based on the initial screening, four isolates (*B. bassiana* WG-21 and WG-18 and *M. anisopliae* WG-07 and WG-02) were selected for further study. The isolate WG-18 was the most virulent against larvae and adults of *B. zonata* and *B. dorsalis* followed by WG-21, WG-02, and WG-07. In both species, adults were more susceptible than larvae to all isolates, and pupae were the least susceptible. Isolates WG-18 and WG-21 strongly decreased female fecundity and fertility, the highest adult and larval mortality, and longest developmental time of larvae and pupae. Fungal conidia were disseminated passively from infected to healthy adults and induced significant mortality, particularly from infected males to non-infected females. In greenhouse and field-cage experiments, WG-18 and WG-21 were the most effective isolates in reducing adult emergence when applied to larvae and pupae of both fruit fly species. Our results indicate that *B. bassiana* isolates WG-18 and WG-21 were the most virulent against multiple life stages of *B. zonata* and *B. dorsalis*, and also exerted the strongest sub-lethal effects.

## 1. Introduction

Fruit flies (Diptera: Tephritidae) are among the most economically important pest species in the world, attacking a wide range of fruits and fleshy vegetables throughout tropical and sub-tropical areas [1,2]. *Bactrocera* Macquart is the most economically significant fruit fly genus with at least 50 species considered to be important pests, many of which are highly polyphagous [2].

Fruit flies cause direct damage to the fruit. Gravid females oviposit just under the fruit skin. Larvae hatch and start feeding on the flesh. Eventually, the fruit rots and prematurely drops to the ground, thereby reducing yield [3,4]. Additional economic losses occur when importing countries prevent the introduction of commodities due to the presence of maggots inside the consignment. As a result, exporting countries lose their potential markets due to quarantine restrictions [5]. In Pakistan and neighboring areas, the oriental fruit fly, *Bactrocera dorsalis* (Hendel), and the peach fruit fly, *Bactrocera zonata* (Saunders) (Diptera: Tephritidae), are considered major threats that limit fruit production.

To combat pestiferous fruit flies, growers largely rely on residual insecticides in the form of direct foliar applications [6] or as bait sprays [7,8] to combat adults, and soil application of insecticides under the tree canopies to kill the fly larvae and pupae. In addition to insecticides, other integrated pest management (IPM) strategies such as the male inhalation technique, parasitoid utilization, cultural practices (i.e., sanitation of orchards), and release of sterile insects have been implemented in other regions of the world including Africa, Japan, Hawaii, and the Pacific region [2,6,9,10]. Nonetheless, due to the repeated and indiscriminate application of insecticides, there are concerns of reduced efficacy through resistance development [11,12,13], and hazards to human health and effects on non-target organisms [14]. This suggests that biologically-based pest control methods could be expanded to achieve more sustainable pest management programs [15].

The potential use of entomopathogenic microorganisms has been explored as a biologically-based treatment strategy to combat insect pests including fruit flies [16,17,18,19,20,21,22]. *Bacillus thuringiensis* strains were found to be somewhat effective but not applicable against tephritid pests due to the insect’s concealed behavior [23]. The failure of baculovirus and protozoa to cause disease against olive fruit flies [24] has prompted researchers to focus on entomopathogenic nematodes [17] and entomopathogenic fungi (EPF). A number of studies have evaluated EPF against Mediterranean fruit fly *Ceratitis capitata* (Wiedemann) [25,26,27], Mexican fruit fly *Anastrepha ludens* (Loew) [28,29,30], European cherry fruit fly, *Rhagoletis cerasi* (L.) [31], western cherry fruit fly *R. indifferens* Curran [32], *B. oleae* (Rossi) [33], and *R. pomonella* (Walsh) [18]. However, very little work utilizing EPF isolates has been conducted on *B. zonata* [34,35,36] *B. (Zeugodacus) cucurbitae* (Coquillett) [34,35], and *B. dorsalis* [37,38].

While EPF are clearly virulent against tephritid fruit flies, significant variation exists among EPF strains and species. Some of the variation can be attributed to their genetic composition, co-evolution with hosts in a particular geographic area, and the host from which they were isolated [5,25,39]. Screening locally sourced EPF to develop potential isolates for field application would be beneficial in terms of relative cost and applicability by growers. The literature reporting on the efficacy of native EPF against *B. zonata* and *B. dorsalis* is scarce. This is the first study focused on the virulence of a broad array of native isolates of EPF against *B. zonata* and *B. dorsalis*.

There are multiple ways to evaluate EPF effectiveness against a given host. Host mortality caused by EPF is the most obvious, but level of horizontal transmission from diseased to healthy insect hosts may be another indicator of effectiveness [35,40]. Unlike bacteria and viruses that must be ingested to cause the infection, fungi can penetrate directly through the host cuticle. Several studies have demonstrated that auto-dissemination of fungus can control different insect pests belonging to different orders including Coleoptera [41], Lepidoptera [42], and Diptera [27,28,35,40]. Along with auto-dissemination, EPF can reduce fruit fly fecundity [5].

The objective of this study, conducted under laboratory, greenhouse, and field cage conditions, was to screen different native isolates of EPF against different developmental stages (third-instar larvae, pupae, adults) of *B. zonata* and *B. dorsalis*. Horizontal transmission of EPF in *B. dorsalis* and their effects on mortality and mycosis of adults was also investigated.

## 2. Materials and Methods

### 2.1. Insect Collection and Rearing

*Bactrocera dorsalis* and *B. zonata* were obtained by mass rearing the insects at the Department of Entomology, University of Agriculture, Faisalabad (Pakistan). The adult colony originated from infested fallen fruits of guava, mango, and citrus collected from different orchards in Punjab, Pakistan. Upon emergence, adults were transferred to screened plastic cages (30 cm × 30 cm × 30 cm). Adult flies were provided with water and an adult diet comprised of sugar and enzymatic yeast (3:1 ratio) [35]. A plastic bottle (500 mL) containing mango juice (Fruita Vitals, Nestle, Lahore) was covered with a lid that had small holes (1 mm in diam.) to collect fruit fly eggs [43]. The collected eggs were transferred to an artificial diet [5]. The larvae were fed on the diet until late third instar and then pupated in containers with soil. The environmental conditions were maintained at 25 ± 1 °C and 60–70% relative humidity (RH) [44].

### 2.2. Fungal Isolates and Their Culturing

The 21 different isolates of entomopathogenic fungi (EPF) evaluated were obtained from culture collections maintained at the Microbial Control Laboratory, Department of Entomology, University of Agriculture Faisalabad (Table 1). These isolates originated from different geographical locations of Punjab, Pakistan [45,46]. All isolates were inoculated on potato dextrose agar (PDA) (BD-Difco) in Petri plates (100 mm), sealed with parafilm, and placed inside an incubator at 25 °C with 14:10 h (light:dark) photoperiod for 7–10 days. After incubation, the dry conidia were harvested with a sterile scalpel and placed inside sterile Falcon tubes (50 mL) with 30 mL of 0.05% Silwet L-77 solution and eight glass beads were added and vortexed for 5 mins to reach homogenization. EPF concentrations were determined by pipetting 10 µL of the suspension on both sides of a hemocytometer and counting conidia under the microscope (400×). Conidia viability was evaluated before tests; 100 µL of each suspension was spread on Sabouraud dextrose agar with 1% yeast (SDAY) media in Petri dishes (60 mm), and incubated at 25 °C, with a 14:10 h (light:dark) photoperiod for 16–18 h. After the incubation period, a cover slip was put on the agar surface to score the germination rate under a microscope, and an average of two counts (about 200 conidia per count) was used for each plate. A conidium was counted as germinated if the germ tube was at least twice as long as the conidium [18,47]. Conidia viability for all EPF isolates was greater than 90%.

### 2.3. Experiment 1. EPF Screening Bioassay against Larvae

The objective of this laboratory study was to evaluate the virulence of 21 isolates of EPF against third-instar larvae of *B. zonata* and *B. dorsalis*. The bioassay arenas consisted of transparent plastic cups (30 mL) that contained 20 g of sterile sandy loam soil (57% sand, 25% silt, 18% clay, pH 7.6, organic matter 0.95%). The soil was collected from the field, subjected to sieving with mesh (2 mm pores), air-dried at 26 °C for 10 days [48], and then autoclaved (Model: SX-500; Brand: Tomy, Japan) at 121 °C for 2 h. One mL of solution containing 1 × 10^9^ viable conidia was pipetted on the top of the soil, and one mL of distilled water was also added to achieve a 10% soil moisture content to maintain field capacity. The cups were covered with lids and gently agitated to ensure uniform distribution of conidia. After EPF application, a single third-instar larva of each fruit fly species was released in each cup on top of the soil and the cup was again covered with a lid. Treatment effects were compared against a control that consisted of 2 mL of 0.05% Silwet L-77 applied to the soil and gently agitated. All experimental cups were placed on plastic trays with wet paper towels, covered with plastic bags to retain moisture, and incubated at 25 °C with a 14:10 h (light:dark) photoperiod [18].

Mortality was assessed on the basis of adult emergence by subtracting the total number of adults that emerged from the total number of larvae originally exposed. The bioassay was terminated four days after the first adult emergence was observed in the control group. Each isolate represents a single treatment, and each treatment consisted of three replications of 20 cups each (a total of 60 cups per treatment). The experiment was repeated twice, thereby resulting in six replications (120 cups) per treatment [18].

### 2.4. Experiment 2. EPF Screening Bioassay against Adults

The same 21 EPF isolates tested against larvae were evaluated against the adult stage of each fruit fly species. For the bioassay, 1 mL of each EPF suspension containing 1 × 10^9^ viable conidia was applied to a glass Petri dish (9 cm diam. × 1.5 cm depth), and the control group received 1 mL of 0.05% Silwet L-77 in distilled water. The plates were shaken on a rotary shaker to cover the entire surface until the solution had evaporated. Twenty adults of *B. zonata* or *B. dorsalis* previously cold immobilized were added to each dish, which was then covered with a lid. Three Petri plates were used for each treatment, with a total of 60 insects for each treatment. Flies were exposed to fungal conidia inside Petri dishes for 1 h [40]. Then, all adults from each plate were transferred to a cage (30 cm × 30 cm × 30 cm) containing water and adult food (sugar and enzymatic yeast at a 3:1 ratio). Each plate represented a single replication, and the whole experiment was conducted two times (total of six replications). Adult mortality was recorded daily until 14 days post-treatment [37]. Environmental conditions were maintained at 25 °C with a 14:10 h (light:dark) photoperiod.

### 2.5. Experiment 3. Dose Response Bioassay against Third Instar Larvae and Adults

The third laboratory experiment evaluated four potential isolates (WG-21, WG-18, WG-07, and WG-02), each at four concentrations (1 × 10^5^, 1 × 10^6^, 1 × 10^7^, 1 × 10^8^ conidia mL^−1^), against third instars and adults of each fruit fly species. These isolates were chosen based on the results of the previous assays. The method of treatment application was the same as in the screening bioassay for both developmental stages. For each fungal isolate, each concentration represented a single treatment, and each treatment consisted of three replications (total 60 insects with 20 individuals used in each replication). For larvae, mortality was assessed on the basis of adult emergence as described above. The bioassay was terminated on the fourth day after the first adult emergence in the control group. Adult mortality was recorded 2, 4, 6, 8, 10, 12, and 14 days post-treatment [18,40]. Similar to the screening bioassays described above, the same environmental conditions were maintained in this bioassay.

### 2.6. Experiment 4. Bioassay against Pupae

This experiment was aimed at evaluating four EPF isolates (WG-21, WG-18, WG-07, and WG-02) against the pupae of both fly species. The bioassay arena was similar to the first screening bioassay except that individual 4–5 days old pupa were buried in the soil at a depth of 3 cm. One mL (1 × 10^7^ and 1 × 10^8^ conidia mL^−1^) of solution was pipetted onto the soil surface and the soil was then mixed as described in Experiment 1. After mixing, pupae (4–5 days old) were buried individually in cups at 3-cm depth [18] and the cups were covered with lids. The control consisted of 2 mL of 0.05% Silwet L-77 applied to the soil surface. The rest of the procedure was the same as described above. Pupae that were unable to emerge as adult flies were considered to have died. Upon emergence, adults were transferred to cages (30 cm × 30 cm × 30 cm) and provided with water and adult food, and mortality was recorded over 10 days [29]. Adult mortality and mycosis were determined on a daily basis, and all dead individuals were removed from the cages each day. Each developmental stage (adult or pupa) was placed inside a plastic Petri dish lined with sterile and moist filter paper (Whatman^®^ Sigma-Aldrich, St. Louis, MO, USA). The dish was wrapped with parafilm and finally incubated at 25 °C to observe the presence of fungal outgrowth [5]. Before putting them into plastic Petri dishes, pupae and adults were surface sterilized with 1% sodium hypochlorite, followed by three rinses with distilled water [29]. Twenty individuals were used for each treatment replicate. There were three replicates for each treatment, and the whole experiment was conducted twice, resulting in six replications (120 individuals were used for each treatment).

### 2.7. Experiment 5. Sub-Lethal Effect on Fecundity and Subsequent Development

Based on results from previous bioassays, effects of a sub-lethal concentration (1 × 10^4^ conidia mL^−1^) of four EPF isolates with the greatest potential (WG-21, WG-18, WG-07, and WG-02) were evaluated on female fecundity (total egg produced by single female), egg fertility (egg successfully hatched), and subsequent developmental stages of *B. zonata* and *B. dorsalis*. For this experiment, 20 adults (10 males and 10 females at 12-day old) of each species were inoculated with either an EPF isolate or 0.05% Silwet L-77 (control) in a similar manner as in previous screening bioassays. After inoculation, the experimental insects were transferred to plastic cages (30 cm × 30 cm × 30 cm) and provided with adult food and water. A device constructed of a 500 mL plastic bottle with small holes (1 mm in diam.) and containing mango juice (Fruita Vitals, Nestle, Lahore, Pakistan) was used for egg-lying [43]. Eggs were collected from each bottle every two days. To measure fecundity, the number of eggs laid per female over 2-day periods was recorded. Adult mortality was recorded daily. To quantify egg fertility, 50 eggs were randomly selected from each replication and placed on Petri dishes (60 mm) filled with 1% of agar. Fertility was determined daily up to seven days by recording the number of larvae that hatched from the egg samples. The plates were incubated at 25 °C, 50–60% RH, and a 14:10 h (light:dark) photoperiod. From each replication, 20 larvae were randomly selected, and they were provided with a larval artificial diet [5] to reach the third instar. Larvae were allowed to pupate in soil in 30 mL cups. Larval duration, larval survival, pupal duration, and adult emergence were recorded.

### 2.8. Experiment 6. Horizontal Transmission Bioassay

Horizontal transmission ability of isolates (WG-21, WG-18, WG-07, and WG-02) was assessed using adults of *B. zonata* and *B. dorsalis*. Four different inoculation combinations were evaluated for each isolate: (1) inoculated male + inoculated female; (2) inoculated male + non-inoculated female; (3) non-inoculated male + inoculated female; and (4) non-inoculated male + non-inoculated female (control). The experimental arenas consisted of glass Petri plates (9 diam. × 1.5 cm depth) applied with 1 mL solution of each EPF (1 × 10^8^ viable conidia mL^−1^). The plates were shaken on a rotary shaker until the suspension evaporated. Inoculated insects (either male or female) were exposed to the conidia suspension. The non-inoculated insects were exposed to a solution that contained 0.05% of Silwet L-77. After inoculation, the different groups were released inside separate cages (30 cm × 30 cm × 30 cm) and were allowed to stay there for 24 h. After 24 h, adults were separated by gender, and placed in individual cages with adult food and water. The mortality was recorded on a daily basis for up to 14 days. Each treatment replicate consisted of 40 adults (20 male and 20 female). The experimental conditions were maintained at 25 °C, 50–60% RH, and a 14:10 h (light:dark) photoperiod. Dead adults were collected from cages daily to avoid cross-contamination [35]. The cadavers were surface-sterilized with a 1% solution of sodium hypochlorite followed by three rinses with distilled water. Then, the cadavers were placed inside plastic Petri plates lined with moist sterile filter paper and sealed with parafilm. These plates were incubated at 25 °C to assess fungus development or mycosis. Each inoculation method was considered as a single treatment with three replications, and the experiment was conducted twice.

### 2.9. Experiment 7. Greenhouse Efficacy Trial

The efficacy of four EPF isolates (WG-21, WG-18, WG-07, and WG-02) was evaluated against third-instar larvae and pupae of *B. zonata* and *B. dorsalis* under greenhouse conditions. Plastic trays were filled with 3 kg of dry sterile sandy loam soil. Approximately 200 mL of water and 100 mL of EPF solution (1 × 10^8^ conidia mL^−1^) were added to maintain field capacity, mixed thoroughly, and then soil was put back into the trays. For the bioassay involving pupae, one group of 50 pupae was buried inside the soil at 3-cm depth. The pupae were evenly distributed throughout the plastic tray. For the bioassay involving larvae, the larvae were released on the top of the soil. For the control group, only 300 mL of distilled water with 0.05% Silwet solution L-77 was used. A fine thin cloth was used to cover the tray and was tightened with rubber bands wrapped around the tray. The experiment was conducted using a randomized complete block design (RCBD) with three blocks for each treatment and the experiment was conducted twice at 25 °C. Treatment efficacy was determined on the basis of adult emergence at 14 days of post-treatment.

### 2.10. Experiment 8. Field-Cage Efficacy Trial

A field-cage experiment was conducted on a local farm in the Lodhran District of Pakistan. A screened (mesh size 1.3 mm × 1.3 mm) wooden cage (1.5 m × 1.5 m × 1.5 m) was positioned under a tree canopy of mango. The cage had a zipper that allowed a person to enter the cage for treatment application, insect introduction, and data recording. For treatment application, conidia were mixed with 0.05% Silwet L-77 solution. The concentration was adjusted to 1 × 10^9^ conidia mL^−1^. The solution (1 L) was applied to the soil using a knapsack sprayer. Treatment application was performed at 18:30 hours to avoid a negative impact of UV light on EPF performance. Soon after treatment application, a group of 250 last instars of each species was released inside the cage. Each EPF isolate represented a treatment, and each treatment had three replicates (=three cages). The experiment was conducted twice; thus, each treatment was replicated six times. Each trial was run using a randomized complete block design (RCBD).

Nine days after the release of third instars, a yellow sticky card (21 cm width × 30 cm length) was installed inside the cage in the top center using a wire. Treatment efficacy was determined by recording the total number of adults caught in the sticky cards from the treatments and control groups. The experiment was terminated at 16 days after the capture of the first adult fly [26].

### 2.11. Statistical Analysis

All statistical analyses were conducted using Minitab software [49]. Mortality (each stage and each species) for the treated group was corrected for control mortality by using the Abbott formula [50] and then the data were subjected to analysis of variance (ANOVA). Whenever appropriate, treatment means were separated with Tukey honest student difference (HSD) test [51] with a significance level of 5%. Control mortality was below 5% in all experiments. Probit analysis was used to determine the LC_50_ and LT_50_ in dose response bioassays for each species in Minitab using log-normal distribution [49]. The developmental data were subjected to ANOVA and means were separated at 5% significance level using Tukey (HSD) test. The field cage data, expressed as captures of adult *B. zonata* and *B. dorsalis* in sticky cards, were also subjected to ANOVA for RCBD, and means were compared with Tukey’s (HSD) test at α = 0.05.

## 3. Results

### 3.1. Experiments 1 and 2. EPF Screening Bioassays against Larvae and Adults

For both fruit species, there was a significant effect of EPF isolates on larval mortality (*B. zonata*: *F*_20, 125_ = 242, *p* < 0.001; *B. dorsalis*: *F*_20, 125_ = 225, *p* < 0.001) and adult mortality (*B. zonata*: *F*_20, 125_ = 265, *p* < 0.001; *B. dorsalis*: *F*_20, 125_ = 217, *p* < 0.001). The 21 EPF isolates tested showed varying levels of virulence, with mean mortality ranging from 2.5% to 88.2% for larvae, and from 5.9% to 100% for adults of *B. zonata* (Table 2). In the case of *B. dorsalis*, except for WG-19, WG-03, and WG-16, all isolates were found to be highly effective against the larvae. The isolates showed variable levels of virulence against adults that in general terms were greater than those recorded for the larvae. Against both *B. zonata* and *B. dorsalis*, the isolate WG-18 was most virulent and caused the highest larval and adult mortality, followed by WG-21, WG-2, and WG-7 (Table 2). The four isolates were thus selected for follow-up bioassays.

### 3.2. Experiment 3. Dose Response Bioassay against Third Instar Larvae and Adults

When tested against larvae, clear dose-dependent mortality was observed among the four different isolates of the EPFs tested. For both fruit fly species, there was a direct positive relationship between mortality and concentration, although no isolate was able to cause 100% mortality. The highest larval mortality was 80.3% for *B. zonata* and 72.8% for *B. dorsalis*, both at the highest conidia concentration. The isolates WG-18 and WG-21 were similarly effective against both fly species at all tested concentrations. Based on 95% fiducial limits, the lowest LC_50_ against larvae was observed in WG-18, followed by WG-21, WG-02, and WG-07 (Table 3).

When tested against adults, significant differences were observed among different concentrations of all isolates for both fly species (*B. zonata*: *F*_3, 23_ ≥ 12.6, *p* < 0.01; *B. dorsalis*: *F*_3, 23_ ≥ 5.00, *p* < 0.01) at 14 days post-treatment. The highest mortality was observed at 14 days post-treatment for WG-18 at the highest conidial concentration, followed by WG-21. For WG-02 and WG-07, no mortality was observed until the second and fourth days post-treatment for all concentrations. Probit analysis revealed that mortality was dose and day dependent among different isolates. Based on 95% fiducial limits, the minimum time and concentration to kill 50% of the tested population was found in WG-18, followed by WG-21, WG-07, and WG-02, only except that WG-21 had the lowest LC_50_ against adult *B. zonata* (Table 4, Table 5 and Table 6).

In general, for all isolates, the adults had lower LC_50_ values than the larvae of both fly species, suggesting that adults were more susceptible than larvae. For both larvae and adults, LC_50_ values were lower in *B. zonata* than *B. dorsalis* (except for LC_50_ in WG-18 against adults), although the differences were not significant based on 95% fiducial limits (Table 4, Table 5 and Table 6).

### 3.3. Experiment 4. Bioassay against Pupae

For both fly species, there were significant differences among treatments (*B. zonata*: *F*_3, 23_ ≥ 32.7; *p* < 0.01 for 1 × 10^7^ conidia mL^−1^ and *F*_3, 23_ = 24.1; *p* < 0.01 for 1 × 10^8^ conidia mL^−1^ and *B. dorsalis: F*_3, 23_ = 38.2; *p* < 0.01 for 1 × 10^7^ conidia mL^−1^ and *F*_3, 23_ = 16.1; *p* < 0.01 for 1 × 10^8^ conidia mL^−1^). Pupae and emerging adults were susceptible to different fungal isolates at both concentrations. For both species, the maximum cumulative mortality was caused by WG-18 at the higher concentration, followed by WG-21 (at the higher concentration), and no significant difference in mortality was observed between WG-18 and WG-21 at the highest concentration (1 × 10^8^ conidia mL^−1^) and also among concentrations (Table 7). A higher level of mycosis was observed for the pupae and adult of both species.

### 3.4. Experiment 5. Sub-Lethal Effect on Fecundity and Subsequent Development

Against both fly species, there were significant differences among treatments in female fecundity (*B. zonata*: *F*_4, 29_ = 80.8, *p* < 0.01; *B. dorsalis*: *F*_4, 29_ = 86.8, *p* < 0.01), percent fertility (*B. zonata*: *F*_4, 29_ = 28.7, *p* <0.01; *B. dorsalis*: *F*_4, 29_ = 15.3, *p* < 0.01), adult longevity (*B. zonata*: *F*_4, 29_ = 70.2, *p* < 0.01; *B. dorsalis*: *F*_4, 29_ = 46.3; *p* < 0.01), larval duration (*B. zonata*: *F*_4, 29_ = 11.2, *p* < 0.01; *B. dorsalis*: *F*_4, 29_ = 13.3; *p* < 0.01), larval survival (*B. zonata*: *F*_4, 29_ = 19.0, *p* < 0.01; *B. dorsalis*: *F*_4, 29_ = 19.3; *p* < 0.01), pupal duration (*B. zonata*: *F*_4, 29_ = 8.79, *p* < 0.01; *B. dorsalis*: *F*_4, 29_ = 11.1; *p* < 0.01), and adult emergence (*B. zonata*: *F*_4, 29_ = 36.1, *p* < 0.01; *B. dorsalis*: *F*_4, 29_ = 25.4; *p* < 0.01). The lowest fecundity per female was observed for WG-18 against both fly species. The lowest fertility was observed for the WG-18 and WG-21 isolates, followed by the WG-02 isolate (Table 8). No significant differences in fertility were observed between the WG-07 isolate and the control. Reductions in adult longevity were observed in *B. zonata* for all isolates compared to the control, and in *B. dorsalis,* all treatments except WG-7 reduced adult longevity. Overall, among all isolates, WG-18 had the strongest sub-lethal effects shown as the lowest female fecundity and fertility, the lowest adult and larval survival, and the longest developmental time of larvae and pupae in both *B. zonata* and *B. dorsalis* (Table 8). The isolate WG-21 was not different from WG-18 except in the fecundity of both species and adult longevity of *B. zonata*.

### 3.5. Experiment 6. Horizontal Transmission Bioassay

Significant differences were recorded in male (*B. zonata: F*_3, 23_ ≥ 131, *p* < 0.001; *B. dorsalis*: *F*_3, 23_ ≥ 113, *p* < 0.001) and female mortality (*B. zonata: F*_3, 23_ ≥ 137, *p* < 0.001; *B. dorsalis*: *F*_3, 23_ ≥ 97.5, *p* < 0.001) for each isolate tested. While the highest mortality was recorded for males and females of both fly species when both genders were inoculated, the treatment did not differ significantly from the combination of non-inoculated females with inoculated males, except for the female mortality of *B. zonata* and *B. dorsalis* exposed to males inoculated with WG-18; thus, horizontal transmission was confirmed (Table 9). Transmission of conidia from inoculated males to non-inoculated females was more pronounced compared to inoculated females to non-inoculated males.

### 3.6. Experiment 7. Greenhouse Efficacy Trial

For both fly species, all treatments reduced adult emergence when applied against third instar larvae (*B. zonata*: *F*_5, 29_ = 44.40, *p* < 0.01; *B. dorsalis*: *F*_5, 29_ = 56.98, *p* < 0.01) compared to the control group. Higher adult emergence was observed when treatments were applied against pupae than larvae (*F*_4, 119_ = 26.98; *p* < 0.01); when applied to pupae, only WG-18 and WG-21 reduced the emergence of *B. zonata* (*F*_5, 29_ = 11.40, *p* < 0.01), while all isolates except WG-7 reduced the emergence of *B. dorsalis* (*F*_5, 29_ = 15.20, *p* < 0.01). The isolates WG-18 and WG-21 had the strongest effect and consistently reduced adult emergence of both flies when either larvae or pupae were treated. Isolates WG-7 and WG-2 showed intermediate levels of efficacy against larvae and minimum efficacy against pupae (Table 10).

### 3.7. Experiment 8. Field-Cage Efficacy Trial

Significant effects of treatments, leading to lower adult emergence (as determined by captures of adult flies on sticky cards), were observed in all treatments against both *B. zonata* (*F*_4, 29_ = 93.52; *p* < 0.01) and *B. dorsalis* (*F*_4, 29_ = 74.79; *p* < 0.01) compared to the control (78.5% for *B. zonata* and 83.1% for *B. dorsalis*). The lowest adult emergence (25.9% for *B. zonata* and 34.3% for *B. dorsalis*) was caused by WG-18, which was not different from the isolate WG-21. Isolates WG-7 and WG-2 showed intermediate results, lower efficacy than WG-18 and WG-21, but were not different from each other (Table 11).

## 4. Discussion

Various EPF isolates caused comparatively high levels of mortality in *B. zonata* and *B. dorsalis*, and adults were more susceptible than larvae, whereas pupae were more resistant than larvae. Among the various isolates of EPFs tested, the *B. bassiana* isolates WG-18 and WG-21 showed higher efficacy compared to the *M. anisopliae* isolates WG-2 and WG-7, and the laboratory findings were confirmed in the greenhouse and field-cage trials. Significant sub-lethal effects were recorded for isolates WG-18 and WG-21 in terms of decreased female fecundity and fertility, decreased adult and larval survival, and longest developmental time of larvae and pupae of both fly species. Horizontal transmission from infected to healthy individuals were found in all isolates and both fly species.

The present study showed that all the tested isolates of EPFs were virulent against last instar larvae and adults of *B. zonata* and *B. dorsalis*. These findings confirmed the susceptibility of *B. zonata* [35,36,52,53] and *B. dorsalis* [37,38,54] to EPF infection in previous reports. The isolates tested here were found to vary in their levels of virulence toward different developmental stages, confirming our hypothesis. Differences in virulence of EPFs have been previously documented by [55], who assessed 20 various isolates of EPFs toward third instar larvae of the Mexican fruit fly, *Anastrepha ludens* (Loew). Among the 20 isolates, 13 isolates caused >87% mortality with LT_50_ values that varied from 1.8 to 6.2 days. In turn, Imoulan and Elmeziane [56] evaluated 15 isolates of *B. bassiana* against *C. capitata* and reported mortality values ranging from 65 to 95%. Variations of virulence of the tested isolates may be attributed to genetic diversity among different isolates that originated from different geographic regions [57], differential immune response [58,59] as well as differences in experimental approaches and conditions.

The screening and dose response bioassays demonstrated that adult flies were the most susceptible stage compared to the larval or pupal stages. In accordance with our findings, Gul et al. [39] found high susceptibility of *B. zonata* adults compared to larvae and pupae when exposed to different fungi (*B. bassiana*, *M. anisopliae*, and *Isaria fumosorosae*). Likewise, Mahmoud (2009) [52] also reported that *B. bassiana*, *M. anisopliae*, and *Lecanicillium muscarium* produced greater mortality in adults of *B. zonata*, followed by larvae and pupae. Hussein et al. (2018) [53] found that the adult stage of *B. zonata* was more susceptible to *B. bassiana* and *M. anisopliae* compared to the larvae and pupae stage. In contrast, results obtained by Yousef et al. [24] with one *M. brunneum* strain indicated 60.0% and 82.3% mortality against adults and larvae of *B. oleae*, respectively. Variation in physiological host range in terms of virulence observed among EPF species and strains could be related to conidial attachment on the cuticle and germination as well as strategies to evade the host’s immune system [60]. For example, low susceptibility of larvae may be attributed to the characteristics of cuticle including its density, thickness, and degree of sclerotization [61]. The cuticles of pre-pupating larvae are more sclerotized compared to the adult, which might be the reason for low susceptibility to fungal infection [5].

Low levels of mortality of pupae caused by EPFs have been documented in various studies against *B. zonata* [36,52,53]. Hussein et al. (2018) [53] observed higher adult emergence of *B. zonata* from 4-day old pupae compared to 1-day old pupae when treated with *B. bassiana* and *M. anisopliae*. Results reported by Beris et al. and Furlong and Pell [40,42] used different EPF isolates, different insect species, and also different application rates. The low susceptibility of pupae recorded in this study may be due to the use of older pupae (with 4–5 days old). Ekesi et al. (2002) [62] found that the pupal susceptibility to *M. anisopliae* was reduced with increased age of pupae of *C. capitata*. The reason behind high susceptibility of younger pupae to fungal infection seems to be due to the softer cuticle of young pupae [62]. Interestingly, high adult mortality was recorded after emergence from treated pupae in this study. These results agree with findings from other studies showing high adult mortality from infected pupae in *C. capitata* and other insect pests of other species [61,62,63]. High levels of *A. ludens* adult mortality were observed when old pupae (2 days before adult emergence) were treated [26].

As expected, in this study, the cadavers produced conidia, which can help regulate the pest population through the production of secondary infection, and may also increase pathogen persistence in the environment [64,65]. Most cadavers showed mycosis even at the pupal stage, as reflected by the conidia present in adults that emerged from the pupae. Some cadavers, however, did not produce conidia outside their bodies. This may be attributed to several factors that inhibit conidia development [47,66]. For example, Shimazu (1994) [66] stated that bacteria found in the hemolymph of the insect might be responsible for the inhibition of fungal sporulation.

Several studies have confirmed the ability of EPFs to be transmitted horizontally. Examples include *A. ludens* [25], *C. capitata* [27,40], *B. zonata*, and *B. cucurbitae* [35], and other insect species such as *Anopheles gambiae* [67] and *Glossina morsitans* [68]. All previous studies had shown significant mortality (e.g., 85–100% in *C. capitata* [24], 69–83% in *B. zonata* and 78–88% in *B. cucurbitae* [35]). In our study, infected males of both species were highly infectious to females. Quesada Moraga et al. (2008) [27] observed that males of *C. capitata* were able to disseminate more conidia to females compared to female-to-male transmission. This may be related to the mating process. Contrary to our study, Sookar et al. (2014) [35] suggest that the female disseminated more conidia to the male compared to male to female against *B. zonata* and *B. cucurbitae* in different pairing combinations.

Previously, Quesada Moraga et al. (2004) [69] observed that *B. bassiana* and *M. anisopliae* reduced fertility and fecundity rates and delayed initial oviposition in *C. capitata*; maximum reductions in fertility and fecundity were detected when *C. capitata* adults were treated with *B. bassiana.* Yee and Lacey (2005) [70] detected that *M. anisopliae* treated *R. indifferens* females laid fewer eggs between 3–7 days post-inoculation. This type of information is scarce in tephritid flies.

## 5. Conclusions

This was the first broad biocontrol screening of native EPF isolates to *B. zonata* and *B. dorsalis*. Our combined findings indicate that *B. bassiana* isolates WG-18 and WG-21 were the most lethal against the larvae, pupae, and adults of *B. zonata* and *B. dorsalis,* and also exerted the strongest sub-lethal effects. Therefore, these two isolates ought to be evaluated under expanded field trials. Applications should be targeted underneath tree canopies to reduce densities of the soil-dwelling stages of fruit flies. This research represents a first step toward the sustainable management of *B. zonata* and *B. dorsalis*; the model can be applied to other fruit fly pests and other pest systems.

## Figures and Tables

**Table 1:** Sources of entomopathogenic fungi that were isolated and evaluated against *B. zonata* and *B. dorsalis*.

Fungi Isolate	Host/Substrate	Geographical Origin (Pakistan)
*Metarhizium anisoplae*	-	-
WG-02	Soil (forests)	Changa Manga
WG-03	*Tribolium castaneum*	Murree
WG-04	Soil (vegetables)	Chichawatni
WG-05	*Rhyzopertha dominica*	Khanewal
WG-06	Soil (forests)	Lal Sohanra
WG-07	Soil (forests)	Bahawalpur
WG-08	*Sitophilus oryzae*	Lodhran
WG-09	*Tribolium castaneum*	Basti Maluk
WG-10	Soil (crop fields)	Rawalpindi
*Beauveria bassiana*	-	-
WG-11	Soil (crop fields)	Lal Sohanra
WG-12	Soil (forests)	Chichawatni
WG-14	Soil (vegetables)	Sheikhupura
WG-15	Soil (forests)	Faisalabad
WG-16	*Tribolium castaneum*	Sargodha
WG-17	*Callosobruchus maculatus*	Gujranwala
WG-18	Soil (forests)	Rawalpindi
WG-19	Soil (vegetable)	Sargodha
WG-20	*Tribolium castaneum*	Gujranwala
WG-21	Soil (fruits)	Lahore
WG-22	*Tribolium castaneum*	Gujranwala
WG-24	Soil (forests)	Jhelum

**Table 2:** Percent mortality (mean ± SEM) of third instar larvae and adults of *B. zonata* and *B. dorsalis* when exposed to 21 different isolates of entomopathogenic fungi at 1 × 10^9^ conidia mL^−1^. Same letters within the column indicate no significant differences between isolates (*p* < 0.05; Tukey HSD test).

Isolate	*B. zonata*		*B. dorsalis*	
	Larvae	Adults	Larvae	Adults
WG-02	75.2 ± 2.4 bc	87.2 ± 2.1 bc	69.3 ± 2.7 bc	80.1 ± 1.7 bc
WG-03	2.5 ± 1.1 o	5.9 ± 2.1 p	1.7 ± 1.1 no	4.3 ± 0.9 n
WG-04	12.9 ± 1.2 lmn	22.4 ± 1.6 mn	9.4 ± 0.9 lmn	18.2 ± 1.9 jkl
WG-05	16.3 ± 1.0 lm	28.5 ± 1.2 lm	12.8 ± 1.7 lm	23.3 ± 1.3 ijk
WG-06	21.4 ± 2.0 jkl	35.4 ± 1.2 kl	17.2 ± 1.2 jkl	31.9 ± 2.0 hi
WG-07	70.2 ± 2.2 cd	81.2 ± 2.7 cd	63.3 ± 2.2 cd	77.6 ± 2.5 bc
WG-08	25.7 ± 1.5 jk	42.3 ± 1.8 jk	22.3 ± 1.8 jk	36.2 ± 1.8 h
WG-09	6.8 ± 1.1 no	14.6 ± 1.4 nop	5.1 ± 0.1 mno	11.2 ± 0.9 lmn
WG-10	45.4 ± 2.2 gh	59.6 ± 1.9 gh	39.3 ± 1.8 gh	55.3 ± 2.1 ef
WG-11	38.5 ± 1.4 hi	54.3 ± 2.1 hi	34.3 ± 1.4 hi	47.5 ± 1.1 fg
WG-12	57.4 ± 3.1 ef	70.9 ± 2.5 ef	52.9 ± 3.3 ef	64.7 ± 2.3 de
WG-14	19.7 ± 1.7 kl	30.2 ± 1.9 lm	13.7 ± 1.8 klm	25.9 ± 2.1 ij
WG-15	15.4 ± 1.4 lmn	26.8 ± 1.7 lm	9.4 ± 1.5 lmn	21.5 ± 1.9 jk
WG-16	3.4 ± 1.1 o	10.3 ± 1.3 op	2.5 ± 1.1 no	8.6 ± 1.11 mn
WG-17	8.6 ± 1.1 mno	17.2 ± 1.6 no	6.9 ± 1.2 mno	14.7 ± 1.7 klm
WG-18	88.2 ± 2.7 a	100.00 ± 0.00 a	80.4 ± 1.8 a	92.3 ± 2.14 a
WG-19	2.5 ± 1.1 o	9.4 ± 1.5 op	0.0 ± 0.00 o	7.8 ± 1.2 mn
WG-20	49.6 ± 2.0 fg	65.7 ± 2.4 fg	45.2 ± 1.6 fg	59.6 ± 2.9 e
WG-21	81.2 ± 1.7 ab	93.1 ± 1.7 ab	76.2 ± 2.3 ab	87.2 ± 2.4 ab
WG-22	64.1 ± 1.9 de	76.0 ± 2.2 de	58.2 ± 2.3 de	71.6 ± 2.8 cd
WG-24	30.00 ± 1.8 ij	48.4 ± 1.9 ij	25.7 ± 1.9 ij	41.4 ± 2.1 gh

**Table 3:** Probit analysis estimates of lethal concentrations required to kill 50% (LC_50_) of larvae of *B. zonata* and *B. dorsalis* along with their 95% fiducial limits. *p* value represents the goodness of fit test.

Fly Species	Isolate	LC_50_ (95% Fiducial Limits)	Slope	Intercept	Chi Square (df = 2)	*p*
*B. zonata*	WG-21	2.5 × 10^6^ (1.2 × 10^6^–5.0 × 10^6^)	0.17 ± 0.02	−7.15	0.29	0.862
	WG-18	1.0 × 10^6^ (2.2 × 10^5^–2.0 × 10^6^)	0.18 ± 0.02	−7.07	0.44	0.799
	WG-07	1.2 × 10^6^ (9.3 × 10^6^–5.7 × 10^7^)	0.16 ± 0.02	−7.44	0.061	0.97
	WG-02	6.1 × 10^6^ (3.0 × 10^6^–1.4 × 10^7^)	0.16 ± 0.02	−7.21	0.20	0.90
*B. dorsalis*	WG-21	9.2 × 10^6^ (4.5 × 10^6^–2.2 × 10^7^)	0.16 ± 0.02	−7.41	0.15	0.92
	WG-18	3.1 × 10^6^ (1.6 × 10^6^–6.4 × 10^6^)	0.17 ± 0.02	−7.20	0.43	0.80
	WG-07	7.3 × 10^7^ (2.8 × 10^7^–3.4 × 10^8^)	0.15 ± 0.02	−7.46	0.12	0.94
	WG-02	2.5 × 10^7^ (1.1 × 10^7^–7.8 × 10^7^)	0.15 ± 0.02	−7.28	0.52	0.77

**Table 4:** Probit analysis estimates of lethal time (days) required to kill 50% (LT_50_) of adult of *B. zonata* along with their 95% fiducial limits. *p* value represents the goodness of fit test.

Isolate	Concentration	LT_50_ (95% Fiducial Limits)	Slope	Intercept	Chi Square (df = 2)	*p*
WG-21	10^5^	12.5 (11.9–13.4)	2.28 ± 0.20	−11.96	0.95	0.96
	10^6^	11.4 (10.9–12.1)	2.20 ± 0.18	−12.71	2.52	0.77
	10^7^	10.4 (9.9–11.1)	1.94 ± 0.15	−13.36	1.33	0.93
	10^8^	8.6 (8.0–9.1)	1.56 ± 0.10	−14.38	25.95	<0.01
WG-18	10^5^	11.0 (10.5–11.7)	2.16 ± 0.17	−12.89	5.75	<0.01
	10^6^	9.8 (9.3–10.3)	2.25 ± 0.16	−13.74	5.52	0.35
	10^7^	8.4 (8.0–8.9)	1.75 ± 0.11	−14.60	21.85	<0.01
	10^8^	7.0 (6.6–7.4)	1.66 ± 0.10	−14.97	36.01	<0.01
WG-07	10^5^	14.7 (13.8–16.1)	2.83 ± 0.32	−9.65	1.04	0.95
	10^6^	13.7 (12.9–14.8)	2.37 ± 0.23	−11.08	1.60	0.90
	10^7^	12.6 (11.9–13.5)	2.23 ± 0.20	−11.98	3.92	0.56
	10^8^	11.7 (11.1–12.4)	2.24 ± 0.18	−12.64	3.97	0.55
WG-02	10^5^	14.9 (13.7–16.6)	2.10 ± 0.22	−10.62	8.67	0.12
	10^6^	13.7 (12.7–15.1)	1.85 ± 0.18	−11.45	9.03	0.10
	10^7^	12.3 (11.5–13.3)	1.88 ± 0.16	−12.19	10.03	0.07
	10^8^	10.6 (10.0–11.3)	1.83 ± 0.14	−13.22	10.64	0.59

**Table 5:** Probit analysis estimates of lethal time (days) required to kill 50% (LT_50_) of adult of *B. dorsalis* along with their 95% fiducial limits. *p* value represents the goodness of fit test.

Isolate	Concentration	LT_50_ (95% Fiducial Limits)	Slope	Intercept	Chi Square (df = 2)	*p*
WG-21	10^5^	13.6 (12.7–14.8)	2.11 ± 0.20	−11.33	1.68	0.89
	10^6^	12.4 (11.7–13.3)	2.24 ± 0.19	−12.11	2.51	0.77
	10^7^	11.4 (10.8–12.2)	1.91 ± 0.17	−12.92	3.66	0.59
	10^8^	10.0 (9.4–10.7)	1.71 ± 0.12	−13.94	28.79	<0.01
WG-18	10^5^	12.3 (11.6–13.2)	2.19 ± 0.19	−12.09	1.53	0.90
	10^6^	11.1 (10.6–11.7)	2.21 ± 0.17	−12.95	3.79	0.57
	10^7^	9.7 (9.2–10.3)	1.92 ± 0.14	−13.75	2.03	0.84
	10^8^	8.5 (8.0–9.1)	1.63 ± 0.11	−14.44	22.43	<0.01
WG-07	10^5^	15.9 (14.6–18.0)	2.30 ± 0.27	−9.55	0.94	0.96
	10^6^	14.4 (13.4–15.8)	2.28 ± 0.23	−10.65	1.09	0.95
	10^7^	13.7 (12.8–14.9)	2.15 ± 0.21	−11.27	2.25	0.81
	10^8^	12.4 (11.7–13.2)	2.25 ± 0.20	−12.15	3.16	0.67
WG-02	10^5^	14.9 (13.8–16.6)	2.24 ± 0.24	−10.35	1.85	0.86
	10^6^	13.8 (12.9–15.0)	2.19 ± 0.21	−11.21	4.78	0.44
	10^7^	12.4 (11.7–13.2)	2.23 ± 0.19	−12.15	4.07	0.53
	10^8^	11.4 (10.7–12.1)	1.93 ± 0.15	−12.97	4.59	0.46

**Table 6:** Probit analysis of estimate lethal concentrations (conidia mL^−1^) required to kill 50% (LC_50_) of adults of *B. zonata* and *B. dorsalis* along with their 95% fiducial limits. *p* value represents the goodness of fit test.

Insect Species	Isolate	LC_50_ (95% Fiducial Limits)	Slope	Intercept	Chi Square (df = 2)	*p*
*B. zonata*	WG-21	1.5× 10^5^ (3.3× 10^2^–8.5× 10^5^)	0.11 ± 0.02	−3.04	1.20	0.54
	WG-18	1.8× 10^5^ (2.2× 10^3^–6.0× 10^5^)	0.18 ± 0.02	−4.36	0.34	0.84
	WG-07	4.6× 10^5^ (2.3× 10^6^–6.9× 10^7^)	0.08 ± 0.02	−3.30	0.05	0.97
	WG-02	9.5× 10^5^ (4.1× 10^3^–4.0× 10^6^)	0.10 ± 0.02	−3.30	1.34	0.51
*B. dorsalis*	WG-21	6.6× 10^5^ (3.1× 10^3^–2.8× 10^5^)	0.10 ± 0.02	−3.41	0.47	0.78
	WG-18	1.7× 10^5^ (6.6× 10^2^–4.8× 10^5^)	0.12 ± 0.02	−3.34	0.33	0.84
	WG-07	1.8× 10^6^ (9.5× 10^6^–2.5× 10^9^)	0.07 ± 0.02	−3.34	0.29	0.86
	WG-02	2.3× 10^5^ (1.15× 10^5^–9.12× 10^5^)	0.09 ± 0.02	−3.26	0.01	0.99

**Table 7:** Mortality and mycosis levels (% mean ± SEM) of the pupae and adults of *B. zonata* and *B. dorsalis* when pupae were exposed to four isolates of entomopathogenic fungi (WG-21, WG-18, WG-07, and WG-02) at two different concentrations (1 × 10^7^ and 1 × 10^8^ conidia mL^−1^). Upper case letters between the rows and lower case letters within the column indicate no significant differences between isolates for mortality and mycosis (*p* < 0.05; Tukey HSD test).

Fly Species	Treatments	Mortality		Mycosis	
		1 × 10^7^	1 × 10^8^	1 × 10^7^	1 × 10^8^
*B. zonata*	WG-21	58.2 ± 3.0 Ab	67.5 ± 3.6 Aa	28.3 ± 2.5 Aa	29.2 ± 3.0 Aab
	WG-18	70.1 ± 3.1 Aa	76.8 ± 3.9 Aa	34.2 ± 2.0 Aa	37.2 ± 1.6 Aa
	WG-07	36.0 ± 2.2 Bc	43.6 ± 1.4 Ab	13.3 ± 1.6 Ab	16.6 ± 2.1 Ac
	WG-02	45.4 ± 2.0 Bc	54.8 ± 2.1 Ab	19.2 ± 2.0 Ab	21.7 ± 1.1 Abc
	*F*_3, 23_	32.7	24.1	20.4	14.3
	*p*	<0.01	<0.01	<0.01	<0.01
*B. dorsalis*	WG-21	52.7 ± 2.8 Bb	63.6 ± 4.0 Aab	25.0 ± 2.6 Ab	30.8 ± 2.4 Aa
	WG-18	67.4 ± 3.4 Aa	71.5 ± 4.0 Aa	33.34.01 Aa	36.7 ± 2.5 Aa
	WG-07	30.2 ± 2.1 Bd	38.7 ± 2.7 Ac	9.2 ± 0.8 Bc	14.2 ± 2.0 Ab
	WG-02	40.6 ± 1.8 Bc	54.3 ± 3.1 Ab	17.5 ± 1.7 Bb	25.0 ± 1.3 Aa
	*F*_3, 23_	38.2	16.1	33.3	10.6
	*p*	<0.01	<0.01	<0.01	<0.01

**Table 8:** Sub-lethal effects of four different isolates (WG-21, WG-18, WG-07, and WG-02) of entomopathogenic fungi tested at 1 × 10^4^ conidia mL^−1^ on the reproduction and development of *Bactrocera zonata* and *B. dorsalis.* Same letters within the column indicate no significant differences between isolates (*p* < 0.05; Tukey HSD test).

Fly Species	Isolate	Fecundity/Female	Fertility (%)	Adult Longevity (Days)	Larval Duration (Days)	Larval Survival (%)	Pupal Duration (Days)	Adult Emergence (%)
*B. zonata*	WG-21	307.7 ± 5.2 d	54.0 ± 3.1 bc	16.3 ± 0.8 d	11.9 ± 0.7 ab	62.7 ± 3.3 cd	12.6 ± 0.8 ab	43.8 ± 3.1 c
	WG-18	275.2 ± 4.4 e	42.3 ± 2.7 c	11.1 ± 0.6 e	12.7 ± 0.6 a	51.3 ± 2.1 d	13.5 ± 0.4 a	34.6 ± 2.5 c
	WG-07	363.8 ± 4.2 b	69.7 ± 3.1 ab	27.3 ± 1.1 b	9.5 ± 0.3 c	76.0 ± 2.8 ab	10.6 ± 0.4 bc	63.4 ± 2.9 b
	WG-02	341.9 ± 5.9 c	61.3 ± 2.9 bc	22.0 ± 0.9 c	10.3 ± 0.3 bc	70.3 ± 3.8 bc	11.7 ± 0.5 abc	55.4 ± 2.0 b
	Control	387.3 ± 4.9 a	80.7 ± 1.8 a	32.1 ± 1.4 a	8.8 ± 0.4 c	83.7 ± 1.7 a	9.5 ± 0.4 c	75.1 ± 2.6 a
*B. dorsalis*	WG-21	326.1 ± 3.8 d	58.0 ± 3.4 cd	18.3 ± 0.9 c	12.2 ± 0.8 ab	66.66 ± 3.37 cd	12.7 ± 0.9 ab	45.2 ± 3.7 c
	WG-18	294.6 ± 5.3 e	49.3 ± 3.4 d	14.1 ± 0.9 c	13.5 ± 0.4 a	57.00 ± 2.90 d	13.9 ± 0.4 a	41.8 ± 2.6 c
	WG-07	385.1 ± 4.3 b	74.3 ± 3.3 ab	31.6 ± 1.7 a	9.6 ± 0.4 c	81.33 ± 2.10 ab	10.8 ± 0.4 bc	67.3 ± 4.1 b
	WG-02	364.0 ± 5.9 c	67.7 ± 4.1 bc	24.2 ± 1.3 b	10.1 ± 0.3 bc	74.00 ± 2.47 bc	12.00 ± 0.6 ab	59.7 ± 1.9 b
	Control	405.7 ± 4.6 a	82.3 ± 2.3 a	35.3 ± 1.5 a	9.4 ± 0.3 c	85.33 ± 1.76 a	9.2 ± 0.1 c	80.6 ± 3.2 a

**Table 9:** Mortality and mycosis levels (% mean ± SEM) caused by horizontal transmission of entomopathogenic fungi (isolates WG-21, WG-18, WG-07, and WG-02) in adults of *B. zonata* and *B. dorsalis* recorded for four different adult pair combinations. Data were collected at 14 days post-inoculation. Upper case letters between the rows and lower case letters within the column indicate no significant differences between isolates for mortality and mycosis in inoculation methods (*p* < 0.05; Tukey HSD test).

Isolate	Pairs		*B. zonata*				*B. dorsalis*			
			Male		Female		Male		Female	
	Male	Female	Mortality	Mycosis	Mortality	Mycosis	Morality	Mycosis	Mortality	Mycosis
WG-21	Inoculated	Inoculated	94.2 ± 2.0 Aa	89.2 ± 3.0 ABa	90.0 ± 3.4 Aa	83.3 ± 3.8 ABa	90.8 ± 3.0 Aa	88.0 ± 2.6 ABa	87.5 ± 3.4 Aa	83.3 ± 3.8 ABa
	Inoculated	Non-inoculated	89.2 ± 4.2 ABa	80.8 ± 3.7 Ba	83.3 ± 3.1 Aa	59.2 ± 3.3 Ab	86.7 ± 4.4 ABa	80.0 ± 5.0 ABa	75.8 ± 3.3 ABa	59.2 ± 3.3 Ab
	Non-inoculated	Inoculated	75.8 ± 3.3 Ab	49.2 ± 3.5 ABb	87.5 ± 3.4 Aa	75.8 ± 2.7 ABa	67.5 ± 4.6 ABb	45.8 ± 3.0 Ab	84.2 ± 3.5 Aa	75.8 ± 2.7 ABa
	Non-inoculated	Non-inoculated	5.8 ± 1.5 Ac	-	3.3 ± 1.1 Ab	-	5.8 ± 1.5 Ac	-	5.0 ± 1.3 Ab	-
			-	37.3	-	14.1	-	39.5	-	14.1
			-	<0.01	-	<0.01	-	<0.01	-	<0.01
WG-18	Inoculated	Inoculated	98.3 ± 1.1 Aa	97.5 ± 1.1 Aa	96.7 ± 1.7 Aa	93.3 ± 2.5 Aa	97.5 ± 1.1 Aa	96.3 ± 1.1 Aa	93.3 ± 2.5 Aa	90.3 ± 2.5 Aa
	Inoculated	Non-inoculated	95.0 ± 2.6 Aa	94.2 ± 2.7 Aa	87.5 ± 2.2 Ab	66.6 ± 4.0 Ab	94.2 ± 2.7 Aa	93.3 ± 3.1 Aa	81.7 ± 3.3 Ab	66.7 ± 4.0 Ab
	Non-inoculated	Inoculated	72.5 ± 2.8 Ab	58.3 ± 4.4 Ab	93.3 ± 2.1 Aab	86.7 ± 2.1 Aa	75.8 ± 3.3 Ab	49.2 ± 3.3 Ab	90.0 ± 1.8 Aab	86.7 ± 2.1 Aa
	Non-inoculated	Non-inoculated	7.5 ± 1.1 Ac	-	4.2 ± 0.8 AC		7.5 ± 1.1 Ac		4.2 ± 0.8 Ac	-
			-	50.4	-	21.7	-	103	-	21.7
			-	<0.01	-	<0.01	-	<0.01	-	<0.01
WG-07	Inoculated	Inoculated	82.5 ± 2.8 Ba	67.5 ± 3.8 Ca	76.7 ± 3.3 Ba	60.0 ± 3.9 Ca	77.5 ± 3.4 Ba	69.2 ± 3.3 Ca	73.3 ± 3.8 Ba	60.0 ± 3.9 Ca
	Inoculated	Non-inoculated	77.5 ± 2.8 Ba	58.3 ± 3.3 Ca	71.7 ± 2.5 Ba	41.7 ± 1.7 Bb	74.2 ± 3.0 Ba	55.0 ± 2.6 Cb	62.5 ± 3.8 Ba	41.7 ± 1.7 Bb
	Non-inoculated	Inoculated	63.3 ± 3.8 Ab	31.7 ± 2.1 Cb	73.3 ± 4.2 Ba	52.5 ± 2.1 Ca	55.8 ± 3.5 Bb	30.0 ± 2.9 Cc	70.0 ± 3.4 Ba	52.5 ± 2.1 Ca
	Non-inoculated	Non-inoculated	6.66 ± 1.1 Ac	-	4.2 ± 0.8 Ab	-	9.2 ± 1.5 Ac	-	5.0 ± 1.3 Ab	-
			-	34.5	-	11.4	-	45.9	-	11.4
			-	<0.01	-	<0.01	-	<0.01	-	<0.01
WG-02	Inoculated	Inoculated	89.16 ± 4.0 ABa	81.7 ± 4.4 BCa	84.1 ± 4.0 ABa	74.2 ± 3.7 Ba	86.7 ± 4.4 ABa	80.8 ± 4.4 BCa	81.7 ± 3.3 ABa	74.2 ± 3.7 Ba
	Inoculated	Non-inoculated	84.16 ± 4.2 ABa	75.0 ± 3.2 Ba	76.7 ± 3.8 ABa	54.2 ± 3.3 ABb	83.3 ± 3.1 ABa	70.8 ± 3.0 Ba	74.2 ± 4.0 ABa	54.2 ± 3.2 ABb
	Non-inoculated	Inoculated	69.16 ± 3.0 Ab	40.0 ± 2.6 BCb	81.7 ± 2.1 ABa	67.5 ± 4.2 Bab	64.2 ± 4.4 ABb	37.5 ± 2.8 ABb	78.3 ± 3.6 ABa	67.5 ± 4.2 Bab
	Non-inoculated	Non-inoculated	7.50 ± 1.1 Ac	-	5.8 ± 0.8 Ab	-	5.0 ± 1.3 Ac	-	4.2 ± 1.5 Ab	-
			-	41.6	-	7.30	-	42.9	-	7.30
			-	<0.01	-	<0.01	-	<0.01	-	<0.01

**Table 10:** Percent adult emergence (mean ± SEM) in *B. zonata* and *B. dorsalis* when four isolates (WG-21, WG-18, WG-07, and WG-02) of entomopathogenic fungi were applied against third instar larvae and pupae in greenhouse trial. Same letters within the column indicate no significant differences between treatments (*p* < 0.05; Tukey HSD test).

Treatment	Larvae		Pupae	
	*B. zonata*	*B. dorsalis*	*B. zonata*	*B. dorsalis*
WG-21	27.3 ± 4.2 bc	32.0 ± 4.3 bc	67.0 ± 4.2 bc	71.3 ± 3.4 cd
WG-18	21.7 ± 4.1 c	25.3 ± 2.9 c	58.7 ± 3.5 c	64.0 ± 2.3 d
WG-07	41.3 ± 4.6 b	45.6 ± 2.8 b	82.3 ± 3.5 a	85.0 ± 2.6 ab
WG-02	34.0 ± 2.5 bc	37.3 ± 2.9 bc	75.7 ± 2.4 ab	78.3 ± 2.0 bc
Control	85.3 ± 2.2 a	87.3 ± 2.2 a	87.3 ± 2.3 a	91.0 ± 2.5 a

**Table 11:** The percentage of adult emergence (mean ± SEM) in *B. zonata* and *B. dorsalis* when four isolates (WG-21, WG-18, WG-07, and WG-02) of entomopathogenic fungi were applied against third instar larvae in a field-cage test. Same letters within the column indicate no significant differences between isolates (*p* < 0.05; Tukey HSD test).

Treatment	*B. zonata*	*B. dorsalis*
WG-21	32.8 ± 1.2 c	41.1 ± 1.5 c
WG-18	25.9 ± 2.0 c	34.3 ± 1.7 c
WG-07	49.6 ± 2.0 b	57.1 ± 2.2 b
WG-02	44.7 ± 1.8 b	53.7 ± 2.6 b
Control	78.5 ± 3.2 a	83.1 ± 2.5 a

## Data Availability

Data are contained within the article.
